# Discrepancies between Coronary Artery Calcium Score and Coronary Artery Disease Severity in Computed Tomography Angiography Studies

**DOI:** 10.3390/diagnostics14171928

**Published:** 2024-09-01

**Authors:** Paweł Gać, Arkadiusz Jaworski, Agnieszka Parfianowicz, Jakub Karwacki, Andrzej Wysocki, Rafał Poręba

**Affiliations:** 1 Centre for Diagnostic Imaging, 4th Military Hospital, 50-981 Wroclaw, Poland; 2 Department of Population Health, Division of Environmental Health and Occupational Medicine, Wroclaw Medical University, 50-556 Wroclaw, Poland; 3 Department of Angiology and Internal Diseases, Wroclaw Medical University, 50-556 Wroclaw, Poland

**Keywords:** coronary artery disease, coronary computed tomography angiography, coronary artery calcium score, coronary artery disease reporting and data system, population risk, disease severity

## Abstract

The aim of this paper is to demonstrate the difference in usefulness of the coronary artery calcium score (CACS) and the full assessment of the severity of coronary artery disease in coronary computed tomography angiography (CCTA) studies. The difference between the population risk of coronary artery disease (CAD) assessed by the CACS and the severity of CAD was demonstrated in images from two CCTA studies. The first image is from a patient with a CACS of 0 and significant coronary artery stenosis. In the native phase of CCTA examination, no calcified changes were detected in the topography of the coronary arteries. In the middle section of the left descending artery (LAD), at the level of the second diagonal branch (Dg2), a large non-calcified atherosclerotic plaque was visible. Mid-LAD stenosis was estimated to be approximately 70%. The second image features a patient with a high CACS but no significant coronary artery stenosis. The calcium score of individual coronary arteries calculated using the Agatston method was as follows: left main (LM) 0, LAD 403, left circumflex (LCx) 207.7, right coronary artery (RCA) 12. CACS was 622.7, representing a significant population risk of significant CAD. In the proximal and middle sections of the LAD, numerous calcified and mixed atherosclerotic plaques with positive remodeling were visible, causing stenosis of 25–50%. Similarly, in the proximal and middle sections of the LCx, numerous calcified and mixed atherosclerotic plaques with positive remodeling were visualized, causing stenoses of 25–50%. Calcified atherosclerotic plaques were found in the RCA, causing stenosis <25%. The entire CCTA image met CAD-RADS 2 (coronary artery disease reporting and data system) criteria. In summary, CACS may be applicable in population-based studies to assess the risk of significant CAD. In the evaluation of individual patients, a comprehensive assessment of CAD severity based on the angiographic phase of the CCTA examination should be used.

CACS was first published by Agatston in 1990, marking a milestone in the detection of coronary calcium [1]. Multi-slice computed tomography (CT) is most used for estimating CACS, with an alternative being the use of electron beam computed tomography. The radiation exposure time is short, with a radiation dose of about 1 mSv. The estimation of the CACS is typically based on non-contrast cardiac CT [2]. Depending on the research and guidelines, the categories of disease progression based on the CACS index may vary. However, the most common classification includes four coronary artery disease risk categories. CACS 0 corresponds to a very low risk of future coronary events (but an absence of calcification does not imply that there is no risk). A CACS 1–100 indicates low significant CAD risk. A CACS 101–400 is moderately increased significant CAD risk, and a CACS >400 is increased probability of significant CAD [3,4].

The CACS is a widely used tool for assessing the risk of significant CAD and its consequences, such as major adverse cardiovascular events (MACE) [5,6]. Moreover, the CACS appears as a strong negative risk factor. Absent and very low scores, such as a CACS of 0 or lower than 10, are associated with a substantial decrease in predicted risk [7,8]. MACE can be defined as the composite occurrence of any of the following events: late cardiac revascularization (coronary artery bypass graft or percutaneous coronary intervention), hospitalization for unstable angina pectoris or heart failure, nonfatal myocardial infarction, and cardiac death or all-cause mortality. A comprehensive meta-analysis of 34,041 cases of symptomatic CAD, examining the association between the risk of MACE and the extent of CAC, demonstrated a significant correlation between these variables. Among 1601 MACE cases, 1443 occurred in patients with a CACS >1, whereas only 158 cases had a CACS of 0. Patients with a CACS > 400 exhibited the highest risk of MACE [9].

The coronary artery disease reporting and data system (CAD-RADS) is a standardized approach for communicating and reporting findings from CCTA. Its purpose is to categorize CAD patients to streamline decision-making regarding subsequent patient management. The CAD-RADS was first proposed in 2016 [10]. This tool has been demonstrated to correlate with the degree of stenosis measured by invasive coronary angiography (ICA) with high diagnostic accuracy, becoming a significant diagnostic tool for CAD [11,12]. The evidence indicates that the CAD-RADS is predictive of the incidence of MACE and all-cause mortality [13]. Notably, integrating the CAD-RADS into clinical practice led to a reduced need for additional testing and cardiology referrals among patients with non-obstructive CAD [14]. According to the current version of CAD-RADS 2.0 from 2022, categories are determined by the severity of stenosis, suggesting appropriate risk and forms of CAD corresponding to it. The CAD-RADS version for patients with stable chest pain is as follows. CAD-RADS 0 is the absence of stenosis in the coronary arteries, i.e., no CAD. CAD-RADS 1 and CAD-RADS 2 indicate non-obstructive CAD (with maximum stenoses of 1–24% and 25–49%, respectively). Maximum stenosis of 50–69%, i.e., moderate CAD, is classified as CAD-RADS 3; and maximum stenosis of 70–99%, i.e., severe CAD, is classified as CAD-RADS 4. CAD-RADS 5 is a total occlusion of the coronary artery [11].

Although a CACS of zero is considered a valuable “negative risk factor” for atherosclerotic cardiovascular disease, superior to many other characteristics evaluated in routine clinical practice [15], clinicians may encounter patients with symptomatic CAD and a CACS of 0. The images presented in Figure 1 concern these types of situations. The most common pathophysiological mechanism in patients with low or 0 CACS who exhibit CAD symptoms involves the presence of a substantial lipid core covered by a thin fibrous cap containing cholesterol and other fats, without calcification detectable by CT. A predisposing factor for this type of atherosclerotic plaque formation is a high concentration of low-density lipoprotein cholesterol (LDL-C), especially over 190 mg/dL [16]. A large-scale study conducted within the CONFIRM (Coronary CT Angiography Evaluation for Clinical Outcomes: An International Multicenter) registry, a multinational database, comprised 10,037 symptomatic patients without previously diagnosed CAD who underwent both CACS and CCTA. The objective of the research was to ascertain the occurrence and severity of IHD among symptomatic patients who exhibited no evidence of coronary artery calcium on CCTA. Among these patients, 51% (*n* = 5128) had a CACS of 0. The stratification and selection of clinical management for patients with a CACS of 0 involve the use of CCTA to assess the degree of stenosis. The findings revealed that among the individuals with a CACS of 0, 16% exhibited some level of CAD on CCTA, 13% presented non-obstructive CAD characterized by stenosis less than 50%. The incidence of stenosis ≥50% was observed in 3.5% of cases, while stenosis ≥70% was noted in 1.4% of cases. [17].

Another type of situation may also occur, i.e., extremely high CACS values, without significant stenosis in the coronary arteries. Such an example is shown in Figure 2. In study by Blaha et al., in the group of 44,052 asymptomatic individuals assessed to evaluate subclinical atherosclerosis, a CACS > 2000 was observed in 1% of the participants. Representative cases from this group were described in this study, which involved a 48-year-old woman and a 51-year-old man, both with type II diabetes and hypertension. The woman had a CACS of 5031, whereas the man had a score of 6420. The extremely elevated CACS reflected a diffuse severe arteriosclerosis. Patients with multiple risk factors like long-term diabetes mellitus are at higher risk of such scenarios [18]. Notably, localized extensive CAC is associated with a stable stage of atherosclerosis and stable plaque, while a more diffuse distribution of CAC over the coronary tree is linked to increased cardiovascular risk [19].

The possibility of discrepancy between the CACS assessment and the CAD-RADS assessment is a result of the CACS definition itself. CACS provides information the amount of calcium rather than its impact on the lumen size. The distribution of calcium may be subintimal or deep and, depending on the physical progression of the disease, may or may not have an impact on the size of the lumen (e.g., deep calcium or adventitial calcium may not impinge on the subintima of the vessel, or positive remodeling of the vessel may minimize the impact of calcium on the luminal size).

To conclude, computed tomography of the coronary arteries provides parameters indicating both the population risk of coronary artery disease and the severity of coronary artery disease in a specific individual. At the same time, it must be remembered that the population risk of disease is the probability of disease estimated statistically on a large group of subjects. In the case of a specific individual in the population, the severity of the disease cannot be assumed based on it. The importance of assessing coronary artery calcium score in population-based studies may suggest that the CACS should be assessed also in other computed tomography examinations, e.g., during chest CT examinations.

## Figures and Tables

**Figure 1:**
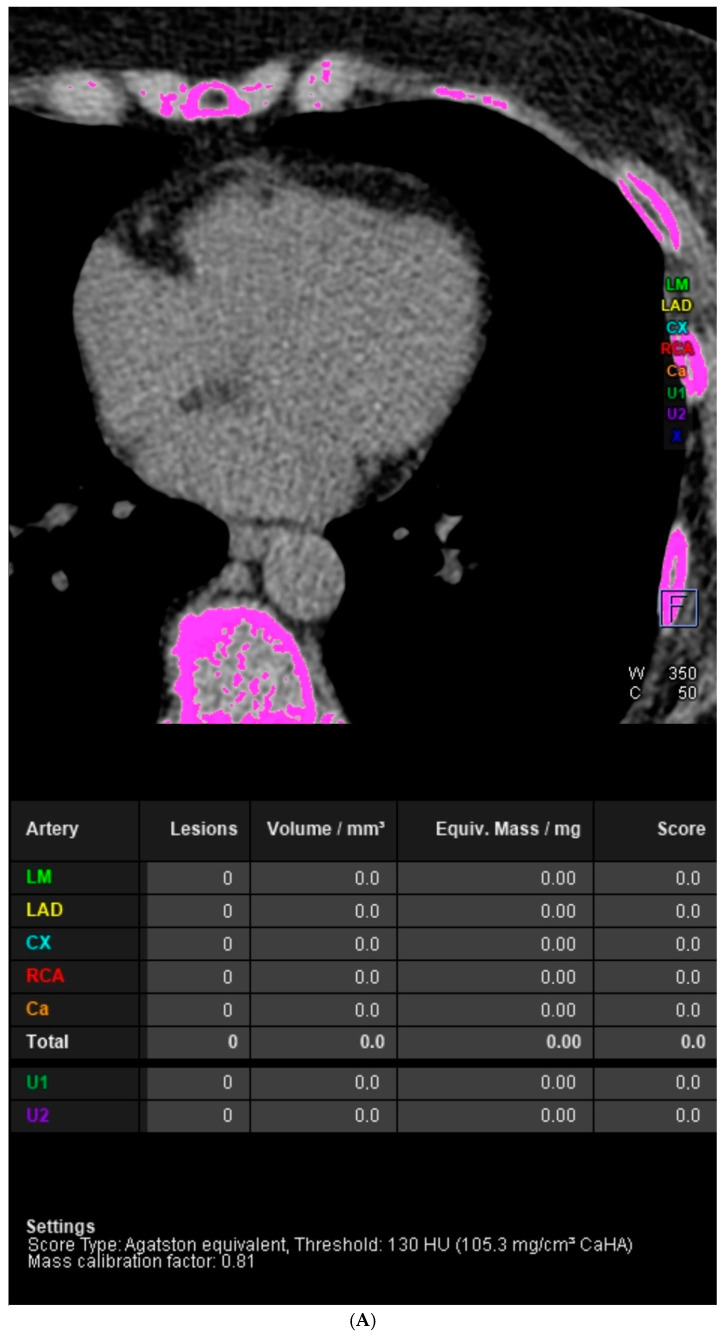

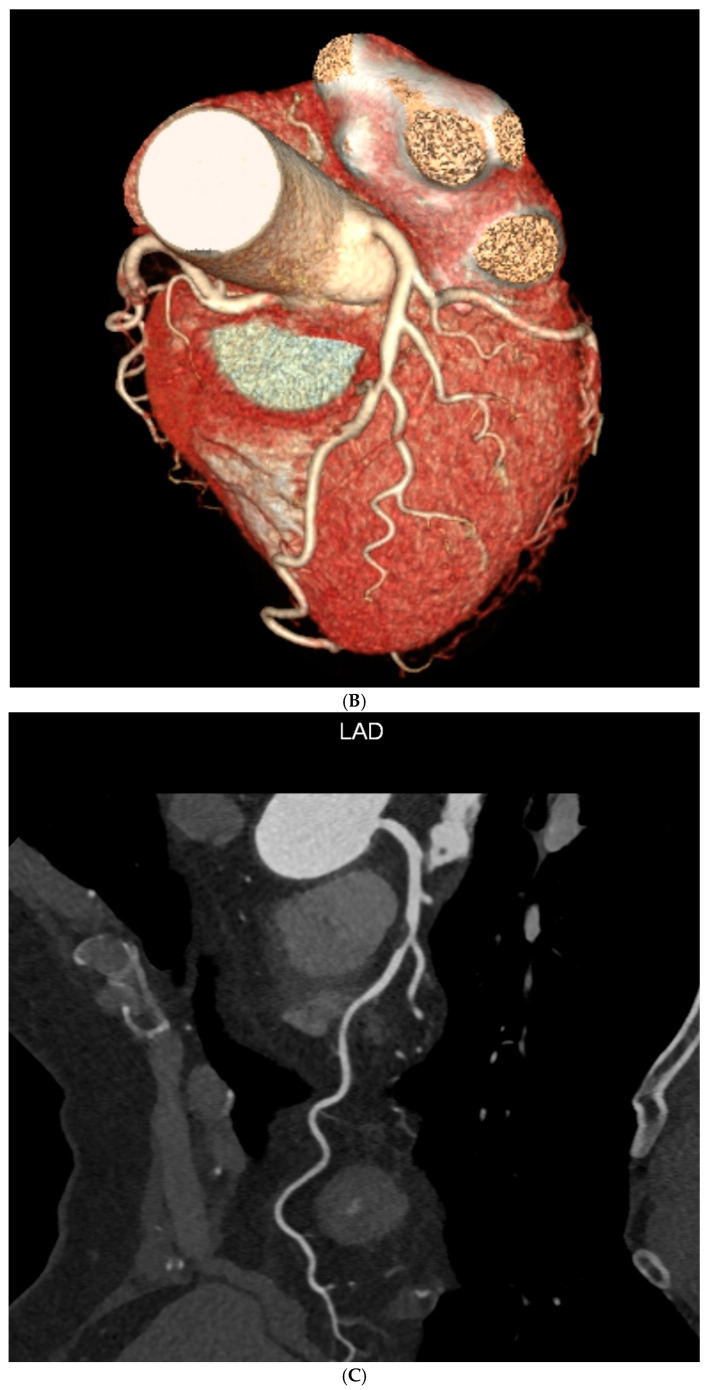

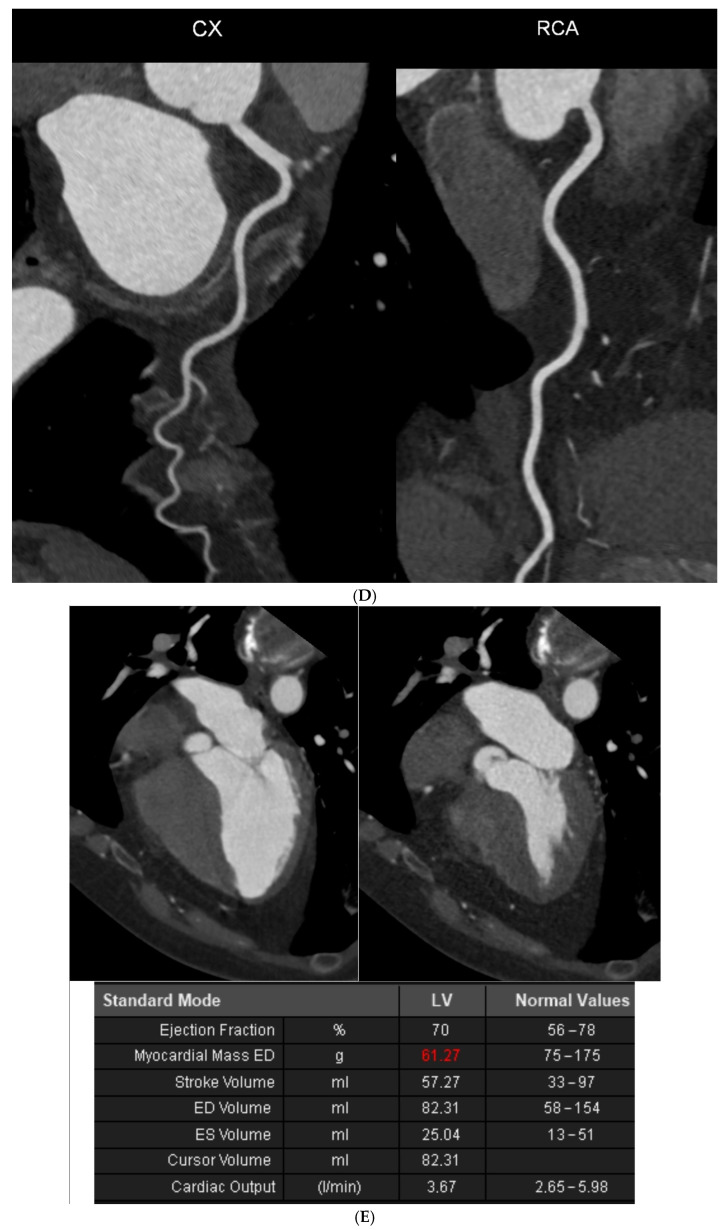
Case 1. Coronary artery calcium score (CACS) = 0 and significant coronary artery stenosis in cardiac computed tomography angiography (CCTA). A 70-year-old female patient was referred for coronary computed tomography angiography via the outpatient cardiology clinic due to reported non-specific chest discomfort and numerous risk factors for cardiovascular diseases in their medical history. CCTA examination was performed according to the standard protocol. (**A**) In the native phase of the CCTA examination, no calcified changes were detected in the topography of the coronary arteries. (**B**) Computed tomography volumetric reconstruction technique. Good-quality images were obtained from the angiographic phase of the CCTA examination. (**C**) Curved multiplanar reconstructions (cMPR). In the middle section of the left descending artery (LAD), at the level of the second diagonal branch (Dg2), a large non-calcified atherosclerotic plaque is visible. Mid-LAD stenosis was estimated to be approximately 70%. The entire CCTA met 4 CAD-RADS (coronary artery disease reporting and data system) criteria.). (**D**) Curved multiplanar reconstructions (cMPR). The left main (LM), left circumflex (LCx), and right coronary artery (RCA) are visualized; these were patent and without stenoses.). (**E**) Assessment of left ventricular functional parameters. The left ventricular ejection fraction was 70%, the left ventricular end-diastolic volume was 82.31 mL, and the left ventricular mass was 61.27 g. The left ventricle was not enlarged and had normal global systolic function. In further diagnostic and therapeutic procedures, invasive coronary angiography confirmed the presence of significant LAD stenosis. Percutaneous coronary intervention with stent implantation in the LAD was performed.

**Figure 2:**
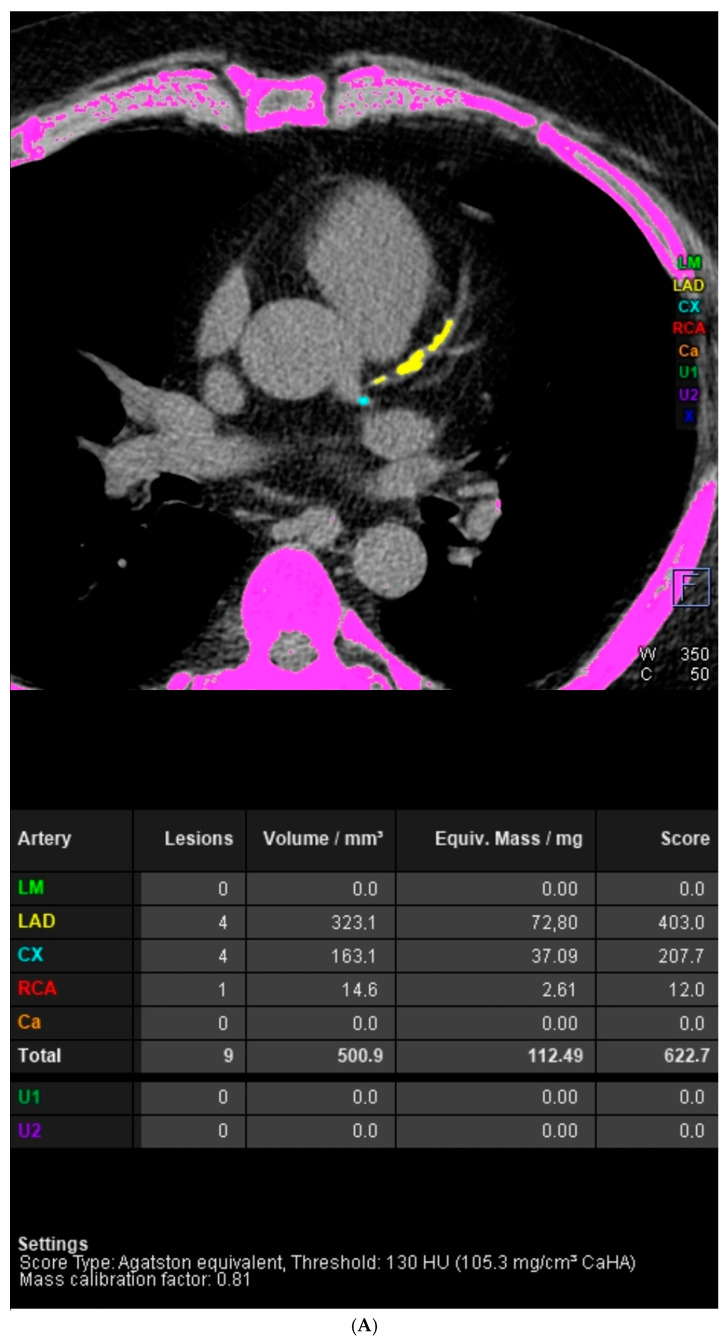

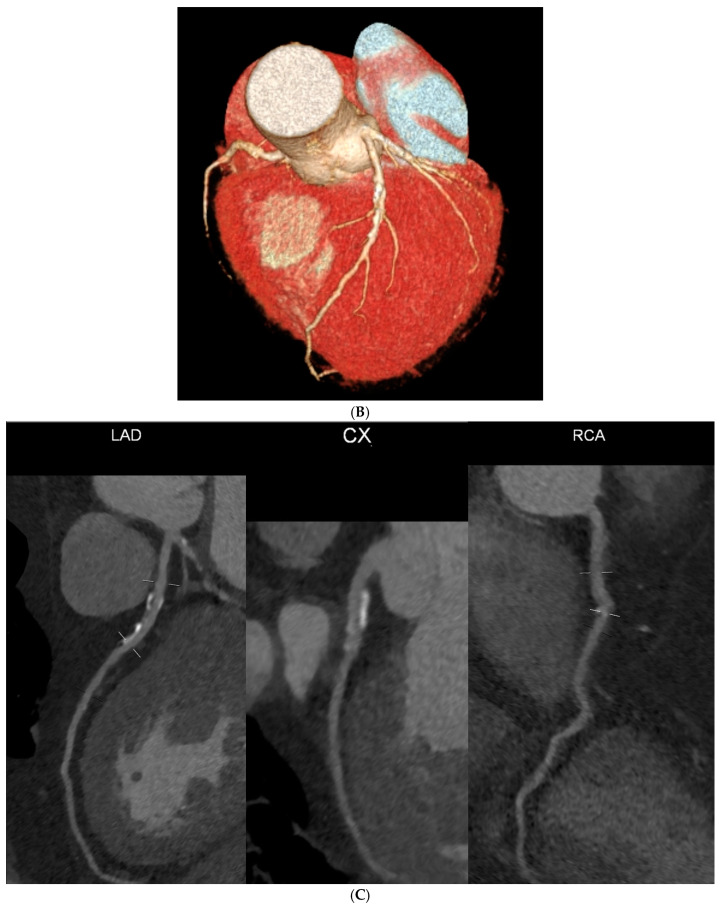

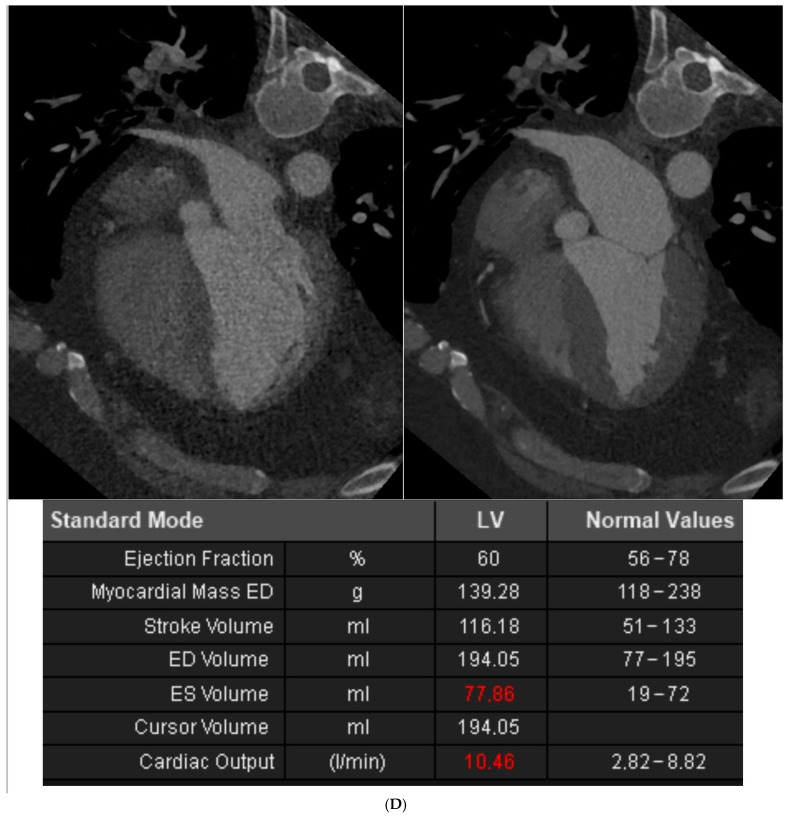
Case 2. A high CACS without significant coronary artery stenosis in CCTA. A 57-year-old male patient was referred for computed tomography angiography of the coronary arteries from the outpatient cardiology clinic due to numerous risk factors for cardiovascular disease in their medical history and sudden cardiac death in the family. CCTA examination was performed according to the standard protocol. (**A**) The native phase of CCTA revealed the presence of calcified changes in the topography of coronary arteries. The calcium index of individual coronary arteries calculated using the Agatston method was LM 0, LAD 403, LCx 207.7, RCA 12. The CACS was 622.7, representing a significant population risk of significant CAD. (**B**) Computed tomography volumetric reconstruction technique. The quality of the obtained images of the angiographic phase of the CCTA examination was assessed as average. The average quality of CCTA images was the result of the patient’s obesity and fast heart rate during acquisition.). (**C**) Curved multiplanar reconstructions (cMPR). LM was short, without stenosis. In the proximal and middle sections of the LAD, numerous calcified and mixed atherosclerotic plaques with positive remodeling were visible, causing stenosis of 25–50%. Similarly, in the proximal and middle sections of the LCx, numerous calcified and mixed atherosclerotic plaques with positive remodeling were visualized, causing stenoses of 25–50%. Calcified atherosclerotic plaques were found in the RCA, causing stenosis <25%. The entire CCTA image met CAD-RADS 2 criteria. (**D**) Assessment of left ventricular functional parameters. The left ventricular ejection fraction was 60%, the left ventricular end-diastolic volume was 194.05 mL, and the left ventricular mass was 139.28 g. The left ventricle was not enlarged and had normal global systolic function. In further diagnostic and therapeutic procedures, due to the occurrence of increasing chest pain, it was decided to verify the coronary arteries via invasive coronary angiography. Invasive coronary angiography excluded significant stenoses in the coronary arteries.

## Data Availability

The data presented in this article are available on request from the corresponding author.

## Abbreviations

CAD	Coronary artery disease;
CCTA	Coronary computed tomography angiography;
CACS	Coronary artery calcium score;
CAD-RADS	Coronary artery disease reporting and data system;
AU	Agatston units;
HU	Hounsfield units;
LDL-C	Low-density lipoprotein cholesterol
ICA	Invasive coronary angiography
